# Complement Factor *C3* Methylation and mRNA Expression Is Associated to BMI and Insulin Resistance in Obesity

**DOI:** 10.3390/genes9080410

**Published:** 2018-08-13

**Authors:** Daniel Castellano-Castillo, Isabel Moreno-Indias, Jose Carlos Fernandez-Garcia, Mercedes Clemente-Postigo, Manuel Castro-Cabezas, Francisco José Tinahones, María Isabel Queipo-Ortuño, Fernando Cardona

**Affiliations:** 1 Unidad de Gestión Clínica de Endocrinología y Nutrición del Hospital Virgen de la Victoria, Instituto de Investigación Biomédica de Málaga (IBIMA), Universidad de Malaga, 29010 Malaga, Spain; 0617617334@uma.es (D.C.-C.); isabel.moreno@ibima.eu (I.M.-I.); josec.fernandez.garcia.sspa@juntadeandalucia.es (J.C.F.-G.); mer.cp@hotmail.com (M.C.-P.); fjtinahones@hotmail.com (F.J.T.); fernando.cardona@ibima.eu (F.C.); 2 Centro de Investigación Biomédica en Red de Fisiopatología de la Obesidad y la Nutrición (CIBERobn), 28029 Madrid, Spain; 3 Department of Internal Medicine Center for Endocrinology, Diabetes and Vascular Medicine St. Franciscus Gasthuis Hospital, 3045 PM Rotterdam, The Netherlands; M.CastroCabezas@franciscus.nl

**Keywords:** DNA methylation, *C3*, ASP, complement factor, obesity, insulin resistance

## Abstract

Epigenetic marks, and especially DNA methylation, are becoming an important factor in obesity, which could help to explain its etiology and associated comorbidities. Adipose tissue, now considered as an important endocrine organ, produces complement system factors. Complement component 3 (*C3*) turns out to be an important protein in metabolic disorders, via either inflammation or the *C3* subproduct acylation stimulating protein (ASP) which directly stimulates lipid storage. In this study, we analyze *C3* DNA methylation in adipose tissue from subjects with a different grade of obesity. Adipose tissue samples were collected from subjects with a different degree of obesity determined by their body mass index (BMI) as: Overweight subjects (BMI ≥ 25 and <30), obese class 1/2 subjects (BMI ≥ 30 and <40) and obese class 3 subjects (BMI ≥ 40). *C3* DNA methylation was measured for 7 CpGs by pyrosequencition using the Pyromark technology (Qiagen, Madrid Spain). *C3* messenger RNA (mRNA) levels were analyzed by pre-designed Taqman assays (Applied biosystems, Foster City, CA, USA) and ASP/C3a was measured using a ELISA kit. The data were analyzed using the statistic package SPSS. *C3* DNA methylation levels were lower in the morbid obese group. Accordingly, *C3* methylation correlated negatively with BMI and leptin. However, *C3* mRNA levels were more associated with insulin resistance, and positive correlations with insulin, glucose and homeostasis model assessment-estimated insulin resistance (HOMA-IR) existed. ASP correlated negatively with high density lipoprotein (HDL) cholesterol. *C3* methylation levels were associated to adiposity variables, such as BMI and leptin, while the *C3* mRNA levels were associated to glucose metabolism.

## 1. Introduction

Obesity is a major public health problem and a significant risk factor for obesity-related diseases, such as type 2 diabetes, hypertension or cardiovascular disease [1]. Adipose tissue is considered a metabolically active immune organ [2]. Human adipose tissue produces and secretes many factors of the complement pathway, complement component 3 (*C3*) being a key molecule in this pathway. It is important to note that *C3* secretion from adipose tissue is proportional to the quantity of adipose tissue, and therefore contributes to the systemic concentrations of this protein [3,4]. C3a, a subproduct of *C3*, is converted in the acylation stimulating protein (ASP), which plays an important role in lipid metabolism by stimulating lipid storage [5,6]. Adipose tissue *C3* production is strongly stimulated in the postprandial state, and in parallel, a peak of ASP at the adipocyte microenvironment level has been shown [7,8,9]. Also, the specific adipocyte factor D (adipsin) is necessary for ASP production. ASP could have a central role in the interplay between the complement system and the metabolism [5]. In this line, obesity has previously been reported as an ASP-resistance situation and plasma levels of *C3* have been associated with obesity [10,11]. Also, serum *C3* is associated with insulin resistance [2], is considered an independent predictor of type 2 diabetes mellitus [2,12], and is a risk marker for cardiovascular disease [3]. 

It is now recognized that epigenetic regulation plays a significant role in complex diseases. Indeed, several studies have associated adipose tissue DNA methylation levels at candidate genes to obesity and metabolic disorders [13,14]. DNA methylation is usually associated with gene silencing and chromatin compaction [15]. DNA methylation regulation may be modified by several nutritional, environmental and metabolic conditions [16,17]. An aberrant DNA methylation pattern (along with changes in gene expression) has been reported in visceral adipose tissue of obese individuals with diabetes [18], suggesting that epigenetic de-regulation can be implied in the etiology of obesity-associated disorders. It has also been reported that the modification of gene expression linked to DNA methylation in subcutaneous adipose tissue is associated to body mass index (BMI) changes, being the modification of gene expression linked to DNA methylation opposite to weight loss and regain of weight [19]. Moreover, a study carried out in twins showed that DNA methylation at regulatory elements specific to adipose tissue had a determinant role in adipose-dependent gene regulation and metabolic disease risk [20].

Thus, the goal of this study is to determine *C3* DNA methylation level in adipose tissue from subjects with different grade of obesity and to study whether *C3* DNA methylation is associated to BMI or obesity-associated disorders.

## 2. Materials and Methods

The study included 60 subjects. Patients were classified according to their BMI as overweight (OW, BMI = 25–29.9 Kg/m^2^), class 1/2 obese (class 1/2, BMI = 30–39.9 Kg/m^2^) and class 3 obese (class 3, BMI ≥ 40 Kg/m^2^). Patients were excluded if they had cardiovascular disease, arthritis, acute inflammatory disease, infectious disease, renal disease or were receiving drugs that could alter the lipid profile or the metabolic parameters at the time of inclusion in the study.

All participants gave their written informed consent and the study was reviewed and approved by the Ethics and Research Committee at Virgen de la Victoria Hospital, Málaga, Spain. All experiments were performed in accordance with relevant guidelines and regulations. ASP was measured in a dimension autoanalyzer (Dade Behring Inc., Deerfield, IL, USA) by enzymatic methods (Randox Laboratories Ltd., Crumlin, UK). Blood analytic variables were measured as was described previously [21]. Homeostasis model assessment-estimated insulin resistance (HOMA-IR) was calculated with the equation HOMA-IR = fasting insulin (μIU/mL) × fasting glucose (mmol/L)/22.5.

Visceral adipose tissue (VAT) samples (omental) were obtained during bariatric surgery or hiatus hernia repair. Biopsy samples were washed in a physiological saline buffer, immediately frozen in liquid nitrogen and maintained at −80 °C until analysis.

### 2.1. RNA Isolation and Gene Expression

Total RNA isolation from adipose tissues was obtained using RNeasy Lipid Tissue Mini Kit (Qiagen, Hilden, Germany) and treated with DNase (RNase-free DNase Set; Qiagen). The RNA concentration was determined by absorbance at 260 nm (A260), and the purity was estimated by determining the A260/A280 ratio with Nanodrop spectrophotometer (NAnodrop Technologies, Wilmington, DE, USA). Pre-made TaqMan assays for Cyclophilin A (4326316E, RefSeq. NM_021130.3) as endogenous control and *C3* (Hs00163811_m1, Refseq. NM_000064.2) were used. Gene expression analyses were carried out as in [21]. Delta threshold cycle (ΔCt) value was calculated by subtracting the Ct value for the corresponding endogenous control complementary DNA from the Ct value for each sample and transcript. Fold changes compared with the endogenous control were then determined by calculating 2^−ΔCt^.

### 2.2. Methylation Analysis DNA

DNA methylation analysis was performed as indicated in [14]. Briefly, 2 µg of genomic DNA isolated from VAT underwent bisulfite conversion using an EpiTect Bisulfite kit (Qiagen). A pre-made Pyromark CpG assay (PM00189399) was used for *C3* DNA methylation analysis, which included seven CpG sites. The polymerase chain reaction (PCR) amplifications were purified using the pyrosequencing Vacuum Prep-Tool (Qiagen, Hilden, Germany), and 15 µL of the PCR products were pyrosequenced using the PyroMarkTMQ96 ID Pyrosequencing System (Qiagen, Hilden, Germany).

The methylation level was expressed as the percentage methylated cytosine over the sum of methylated and unmethylated cytosines. The values are expressed as the mean for the seven sites for the *C3* gene. Inter-assay precision (% coefficient of variation (CV) was <2.5%, intra-assay (%CV) was <1.0%.

### 2.3. Statistical Analysis

Statistical analyses were performed using the SPSS statistical package (version 19 for Windows; SPSS, Chicago, IL, USA). Analysis of variance (ANOVA) with Duncan and Tukey post-hoc analyses were performed to compare means among groups and Pearson’s correlations to assess the associative study. Linear regression models were created to analyze the association of DNA and mRNA *C3* levels with BMI and HOMA-IR. Values were considered to be statistically significant when *p* ≤ 0.05. Figures were performed with GraphPadPrisma (GraphPad Software Inc. version 5.01 for Windows; La Jolla, CA, USA).

## 3. Results

Biochemical and anthropometric characteristics of each study group are summarized in Table 1.

**Figure 1:**
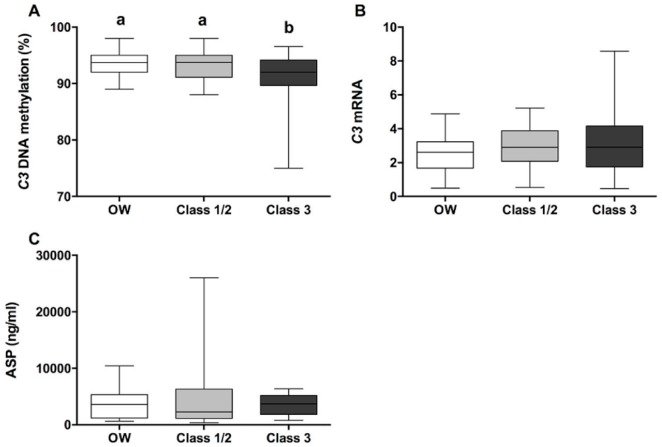
Figure shows the adipose tissue *C3* DNA methylation levels (**A**), adipose tissue *C3* messenger RNA (mRNA) levels (**B**) and serum levels of acylation stimulating protein (ASP) (**C**) among the study groups: OW (overweight subjects; Body mass index (BMI) = 25–29.9 Kg/m^2^), Class 1/2 (class 1/2 obese subjects; BMI = 30–39.9 Kg/m^2^) and Class 3 (class 3 obese subjects; BMI ≥ 40 Kg/m^2^). Values are presented as the means ± standard deviation (SD). Analysis of variance (ANOVA) and post hoc analysis using Duncan and Tukey test was used to test differences among the groups. Different letters mean significant differences among the groups when *p* < 0.05.

*C3* DNA methylation levels were lower in class 3 patients compared to the other study groups (Figure 1A). However, this result was not translated into *C3* mRNA expression (Figure 1B). No differences were found for serum ASP values among the study groups either (Figure 1C).

Biochemical and anthropometric characteristics were related with *C3* DNA methylation, *C3* mRNA expression and serum ASP (Table 2). Interestingly, while *C3* methylation correlated negatively with variables associated to adiposity, such as BMI and serum leptin, *C3* mRNA expression showed a positive correlation with glucose, insulin and HOMA-IR and a negative association with serum adiponectin levels. When the correction for age was made, the correlation seen between BMI and *C3* DNA methylation was still maintained (r = −0.353, *p* = 0.006), while the association present between HOMA-IR and *C3* mRNA levels showed no statistical significance (r = 0.252, *p* = 0.052). In addition, we have observed a positive correlation between *C3* mRNA expression in VAT and the ASP levels (r = 0.7, *p* = 0.034) only in the class 3 group. Finally, we observed a negative association between ASP serum levels and high density lipoprotein (HDL) cholesterol (Table 2).

These relationships, *C3* methylation with BMI and *C3* mRNA with insulin resistance, were reinforced by linear regression analyses. Thus, in a model with BMI as a dependent variable and corrected with variables strongly related to obesity as age or HOMA-IR, *C3* DNA methylation reached a strong significance in the model, which was able to explain up to 48% of the BMI variability (Table 3A). Furthermore, when HOMA-IR was considered as the dependent variable in a multiple lineal regression analysis, *C3* mRNA levels and BMI could explain up to 35% of the variability present in HOMA-IR (Table 3B).

## 4. Discussion

In the current study, we have demonstrated for the first time that class 3 subjects present a lower *C3* DNA methylation, and that *C3* DNA methylation shows a positive association with adiposity parameters (BMI and leptin). On the other hand, *C3* mRNA levels were related to insulin resistance and glucose homeostasis, which is in line with previous results from our group [22] and from other authors [23]. Thus, no differences in *C3* mRNA expression were found among the studied groups, which have already been tested in visceral adipose tissue [24]. 

However, an association of *C3* DNA methylation and *C3* mRNA with ASP was not found, possibly due to the fact that *C3* is mainly produced by the liver, and to a lesser extend in muscle and adipose tissue [25]. However, *C3* is not only produced, but also activated in human adipose tissue. In fact, complement activation products may actually play a role in metabolic events related to a fat mass increase [3,24].

*C3* DNA methylation and mRNA expression were not significantly associated in our results. However, it would be possible that this association could be found at a postprandial level but not at basal level. Indeed, other studies have pointed out a possible response of adipose tissue *C3* production to feeding [7,8,9]. Furthermore, this response is not detectable at plasma levels [26,27,28]. Instead, high levels of *C3* at the adipose tissue microenvironment have been proposed, which, with the help of adipsin (specifically produced in adipose tissue), could produce a peak of ASP at postprandial levels [8,27]. This peak of ASP could stimulate adipocyte to store lipids. Lower levels of *C3* DNA methylation could deregulate this response to feeding, prompting to a pro-inflammatory state [27]. On the other hand, we have described a positive correlation between *C3* mRNA expression in VAT and ASP levels only in the class 3 group. This phenomenon could explain why these class 3 subjects present a higher HOMA-IR. 

Finally, we have observed a significantly positive association between *C3* mRNA and insulin resistant parameters (HOMA-IR, plasma glucose levels and insulin). In line with these results, the effect of plasma *C3* on insulin resistance has been previously reported [12]. Plasma *C3* levels, which could show a metabolic deregulation, may induce insulin resistance progression, eventually leading to type 2 diabetes mellitus [12]. At the same time, insulin resistance could be stimulated by pro-inflammatory cytokines that are able to produce a low chronic inflammatory grade [29]. In turn, this meta-inflammation could be responsible for the activation of the complement system, a situation that could contribute to the deterioration of metabolic complications observed in obesity [30,31,32]. 

We also found a negative association between ASP and HDL cholesterol. ASP plays an important role in lipid metabolism of adipose tissue, acting as a hormone involved in lipid storage and energy homeostasis. Thus, *C3* up-regulation in adipose tissue leads to higher macrophage infiltration, a failure in lipid processing and insulin resistance [33]. We observed lower levels of total cholesterol and HDL cholesterol in the class 3 obese group. HDL cholesterol is a potent anti-inflammatory particle [20,34], while cholesterol accumulation in adipose tissue macrophages has been associated to inflammation and metabolic deterioration [20]. In addition to the known action of ASP over lipid metabolism, this negative association would be in line with previous literature given the anti-inflammatory effect of HDL cholesterol [34].

Several limitations must be taken into consideration in the present study. class 3 group age is slightly lower than the rest of the groups. This fact could be related with methylation levels. However, aging has been associated to a decrease in global DNA methylation [35,36], reinforcing our find about the deregulation of *C3* DNA methylation in these class 3 patients. This would agree with other results where BMI has been associated to epigenetic marks [13], whose modulation could result in new therapeutic strategies [37].

Our results support the fact that the DNA methylation of *C3* is strongly associated to BMI and that *C3* mRNA associates with the pathophysiology of obesity-related metabolic diseases. More studies are necessary to discern the possible role of *C3* DNA methylation in adipose tissue physiology, especially in response to feeding and lipid processing.

## Figures and Tables

**Table 1:** Biochemical and anthropometric variables for each study group.

	Overweight (*n* = 23)	Class 1/2 (*n* = 20)	Class 3 (*n* = 17)
Age (years)	55.70 ± 11.71 a	56.70 ± 15.24 a	41.53 ± 9.78 b
Gender (men/women)	10/13	7/13	6/11
BMI (kg/m^2^)	27.41 ± 1.29 a	33.23 ± 2.76 b	49.78 ± 6.49 c
Waist (cm)	93.96 ± 5.48 a	106.75 ± 9.23 b	133.69 ± 17.77 c
Glucose (mmol/L)	6.12 ± 1.20	5.99 ± 1.47	5.79 ± 1.15
HOMA-IR	2.318 ± 0.98 a	3.124 ± 1.59 a	5.30 ± 4.91 b
Tg (mmol/L)	1.42 ± 0.60	1.47 ± 0.54	1.37 ± 0.50
Cholesterol (mmol/L)	5.58 ± 1.00 a	5.48 ± 1.28 a	4.67 ± 0.94 b
HDL-cho (mmol/L)	1.37 ± 0.34 a,b	1.49 ± 0.32 a	1.26 ± 0.32 b
SBP (mmHg)	131.43 ± 22.37	135.65 ± 24.83	136.07 ± 19.39
DBP (mmHg)	79.52 ± 12.62	79.75 ± 12.78	83.79 ± 10.03
ApoA1 (mmol/L)	1.73 ± 0.20	1.81 ± 0.27	1.58 ± 0.15
ApB (mmol/L)	1.10 ± 0.27	1.01 ± 0.21	0.89 ± 0.28
GOT (µkat/L)	0.25 ± 0.12 a	0.34 ± 0.18 a,b	0.39 ± 0.16 b
GPT (µkat/L)	0.59 ± 0.24	0.75 ± 0.37	0.75 ± 0.32
GGT (µkat/L)	0.63 ± 0.50	0.61 ± 0.29	0.55 ± 0.38
Leptin (ng/mL)	14.31 ± 7.13 a	21.83 ± 11.12 a	68.21 ± 30.09 b
Adiponectin (ng/mL)	9.21 ± 3.92	10.49 ± 4.64	6.90 ± 3.83

Homeostatic model assessment of insulin resistance (HOMA-IR); Triglycerides (Tg); High density lipoprotein cholesterol (HDL-cho); Systolic blood pressure (SBP); Diastolic blood pressure (DBP); Apolipoprotein A1 (ApoA1); Apolipoprotein B (ApoB); Glutamil oxaloacetate transaminase (GOT); glutamate pyruvic transaminase (GPT); Gamma-Glutamyl Transferase (GGT). Different letters mean significant differences between groups (*p* < 0.05).

**Table 2:** Correlations between *C3* DNA methylation, *C3* mRNA and serum ASP with anthropometric and biochemical variables.

	*C3* mRNA	*C3* Methylation	ASP
Age	0.076	0.226	0.035
BMI	0.177	−0.411 **	−0.04
Waist	0.192	−0.26	0.062
Insulin	0.364 **	−0.079	0.157
Glucose	0.324 *	0.09	−0.102
HOMA-IR	0.417 **	−0.079	0.124
HDL-cho	0.073	−0.176	−0.370 **
Leptin	0.289	−0.528 **	−0.077
Adiponectin	−0.316 *	0.005	−0.071

* and ** Indicates differences between the groups (*p* < 0.05 and *p* < 0.01 respectively).

**Table 3:** Multiple regression analysis. Model A with BMI as dependent variable and model B with HOMA-IR as dependent variable. Model A was age-, gender-, HOMA-IR-, *C3* mRNA- and *C3* methylation-adjusted. Model B was age-, gender-, BMI-, *C3* mRNA- and *C3* methylation-adjusted.

**A**	**BMI (R = 0.69, R^2^ = 0.48)**		
	β	*p*	CI (95%)
Age	−0.28	0.00	−0.43–(−0.13)
Gender	0.48	0.81	−3.76–4.73
HOMA-IR	1.12	0.00	0.38–1.87
*C3* mRNA	0.34	0.65	−1.17–1.86
*C3* methylation	−0.79	0.00	−1.35–(−0.23)
**B**	**HOMA-IR (R = 0.59, R^2^ = 0.35)**		
	β	*p*	CI (95%)
Age	−0.00	0.96	−0.06–0.05
Gender	−0.71	0.32	−2.14–0.72
BMI	0.13	0.00	0.04–0.21
*C3* mRNA	0.74	0.00	0.26–1.22
*C3* methylation	0.07	0.44	−0.12–0.28

CI: Confidence Interval.
